# Inhibition of *Staphylococcus epidermidis* Biofilm Formation by Traditional Thai Herbal Recipes Used for Wound Treatment

**DOI:** 10.1155/2012/159797

**Published:** 2012-08-07

**Authors:** S. Chusri, K. Sompetch, S. Mukdee, S. Jansrisewangwong, T. Srichai, K. Maneenoon, S. Limsuwan, S. P. Voravuthikunchai

**Affiliations:** ^1^Faculty of Traditional Thai Medicine, Prince of Songkla University, Hat Yai, Songkhla 90110, Thailand; ^2^Natural Products Research Center, Prince of Songkla University, Hat Yai, Songkhla 90110, Thailand; ^3^Department of Microbiology, Faculty of Science, Prince of Songkla University, Hat Yai, Songkhla 90110, Thailand

## Abstract

Development of biofilm is a key mechanism involved in *Staphylococcus epidermidis* virulence during device-associated infections. We aimed to investigate antibiofilm formation and mature biofilm eradication ability of ethanol and water extracts of Thai traditional herbal recipes including THR-SK004, THR-SK010, and THR-SK011 against *S. epidermidis*. A biofilm forming reference strain, *S. epidermidis* ATCC 35984 was employed as a model for searching anti-biofilm agents by MTT reduction assay. The results revealed that the ethanol extract of THR-SK004 (THR-SK004E) could inhibit the formation of *S. epidermidis* biofilm on polystyrene surfaces. Furthermore, treatments with the extract efficiently inhibit the biofilm formation of the pathogen on glass surfaces determined by scanning electron microscopy and crystal violet staining. In addition, THR-SK010 ethanol extract (THR-SK010E; 0.63–5 **μ**g/mL) could decrease 30 to 40% of the biofilm development. Almost 90% of a 7-day-old staphylococcal biofilm was destroyed after treatment with THR-SK004E (250 and 500 **μ**g/mL) and THR-SK010E (10 and 20 **μ**g/mL) for 24 h. Therefore, our results clearly demonstrated THR-SK004E could prevent the staphylococcal biofilm development, whereas both THR-SK004E and THR-SK010E possessed remarkable eradication ability on the mature staphylococcal biofilm.

## 1. Introduction

*Staphylococcus epidermidis*, a normal inhabitant of the healthy human skin and mucosal microbial communities, has emerged as a common cause of numerous nosocomial infections, mostly occurring in immunocompromised hosts or patients with implanted medical devices [1]. Even though, it has a low pathogenic potential, data from the European surveillance indicated that coagulase-negative staphylococci were isolated up to 40% in bloodstream infections while *Staphylococcus aureus* was isolated less than 20% [2]. In *S. epidermidis*, biofilm formation is regarded as a major pathomechanism as it renders *S. epidermidis* highly resistant to conventional antibiotics and host defenses. This can be caused by slow diffusion of these compounds through the extracellular polymeric matrix and slow growth of the bacteria [3, 4]. Staphylococcal biofilm is therefore difficult to eradicate and is a source of many recalcitrant infections. As such, novel strategies or more effective agents exhibiting an antibiofilm ability with clinical efficacy and safety are of great interest.

Medicinal plant-derived compounds have increased widespread interest in the search of alternative antibacterial agents because of the perception that they are safe and have a long history of use in folk medicine for the treatment of infectious diseases [5]. The active constituents isolated from medicinal plants have intensively been studied for their antibacterial effects against planktonic bacteria. More importantly, some plants have been reported to be able to prevent the formation of biofilm in some pathogens such as *Listeria monocytogenes* [6], *Pseudomonas aeruginosa *[7], *Streptococcus pyogenes *[8], *Streptococcus mutans *[9], and* S. aureus *[10]. So far, most studies have focused on the observation of anti-biofilm activity of herbs taken as a single unit but not in combination, such as in herbal recipes. No attention has been paid to the antibacterial or anti-biofilm activity of traditionally used herbal recipes.

This study was therefore undertaken to investigate the *in vitro* anti-biofilm potential of selected Thai traditional herbal recipes (THRs) that have been traditionally employed for the treatment of wounds and skin infections against an important biofilm producing pathogen, *S. epidermidis*. The biofilm formation requires bacterial attachment to solid surfaces, the development of bacterial multilayers, and their enclosure in a large exopolymeric matrix [3]. Because of that, we investigated both anti-biofilm development and mature biofilm eradication of the recipes.

## 2. Material and Methods

### 2.1. Bacterial Strains and Their Biofilm-Forming Ability

Bacteria used in this study were clinically isolated methicillin resistant *Staphylococcus aureus* (MRSA) NPRC R001-R005, methicillin susceptible *S. aureus* (MSSA) NPRC S001-S005, *S. aureus* ATCC 25923, a biofilm-positive strain (*Staphylococcus epidermidis* ATCC 35984), and a biofilm-negative strain (*S. epidermidis* ATCC 12228). 

In order to assess the biofilm formation ability of *Staphylococcus* spp., well-isolated colonies grown overnight at 37°C on tryptic soy agar (TSA, Becton, Dickinson, and Company, France) were inoculated in tryptic soy broth (TSB, Becton, Dickinson, and Company) supplemented with 2% (w/w) D-glucose (TSBGlc). Following incubation at 37°C for 24 h; culture supernatants from each isolate were diluted 1 : 200 in TSBGlc. Aliquots of bacterial suspension (200 *μ*L; 5 × 10^5^ CFU/mL, final concentration) were transferred into a flat-bottomed 96-well polystyrene microtiter plate (Nunc, Roskilde, Denmark). The medium without the bacterial suspension was used as the negative control. The plates were incubated at 37°C for 24 h, culture supernatants from each well were then decanted and planktonic cells were removed by washing three times with phosphate-buffered saline (PBS; pH 7.4) [10].

3-(4,  5-dimethyl-2-thiazolyl)-2,  5-diphenyl-2H-tetrazolium-bromid  (MTT)  (Sigma-Aldrich,  USA) reduction assay according to the method previously described [11] was applied to quantify the biofilm forming ability of the isolates. An aliquot of MTT solution (0.2 mg/mL; 200 *μ*L) was added to each of the prewashed wells and the plate was then incubated for 3 h in the dark at 37°C. Following the incubation, MTT was then replaced by 200 *μ*L of dimethylsulfoxide (DMSO; Merck, Darmstadt, Germany). Bacteria with an active electron transport system will reduce the tetrazolium salt to a water-insoluble purple formazan product. The colour intensity of DMSO dissolved formazan was determined by a microplate reader (Tecan Sunrise, Tecan Austria) at A482 nm. The absorbance values for the negative control were then subtracted from the tested wells to eliminate false results due to background interference. 

### 2.2. Preparation of Herbal Recipes

Selected southern Thai herbal recipes that have been used for the treatment of wounds and skin infections were kindly provided by Mr. Earn Thongsongsi (THR-SK004) [12] and Mr. Somporn Chanwanisakul (THR-SK010 and THR-SK011), a traditional Thai Medical Doctor at Traditional Thai Medicine Hospital, Prince of Songkla University, Hat Yai, Thailand. Plant parts as described in Table 1 were locally collected and reference voucher specimens were deposited at Faculty of Traditional Thai Medicine, Prince of Songkla University, Hat Yai, Songkhla, Thailand. The powdered formulas (100 g) were submitted to solvent extractions by maceration with distilled water at room temperature for three days or with ethanol for seven days (500 mL each solvent). After filtrations through a Whatman no. 1 paper, aqueous filtrates were freeze-dried and ethanol filtrates were concentrated using a rotatory evaporator, and kept at 55°C until they were completely dry. Yields (%; w/w) of each extracts were calculated as the ratio of the weight of the extract to the weight of the crude herb powder and presented in Table 1. Lyophilized water extracts (THR-SK004W, THR-SK010W, and THR-SK011W) and evaporated ethanol extracts (THR-SK004E, THR-SK010E, and THR-SK011E) were dissolved in 10% dimethylsulfoxide (DMSO; Merck, Germany) before use.

### 2.3. Inhibition of Staphylococcal Biofilm Formation by the Herbal Recipes. 

The herbal recipe extracts were tested for their potential to prevent biofilm formation of a biofilm producing strain, *S. epidermidis *ATCC 35984. They were added to the growth medium at the time of inoculation and the cells were allowed to form biofilm. An aliquot of twofold serial dilutions (100 *μ*L) was prepared in the 96-well microtiter plate containing TSBGlc, with final concentrations of THR-SK004W, THR-SK010W, THR-SK011W, THR-SK004E, and THR-SK011E ranging from 15.63 to 2501 *μ*g/mL and 0.63 to 250 *μ*g/mL for THR-SK010E. Bacterial suspensions (100 *μ*L; 5 × 10^5^ CFU/mL, final concentration) were then transferred into the plate. TSBGlc containing 0.2% DMSO was employed as a negative control. TSBGlc without the extract was used as the nontreated well and the medium with each concentration of the extracts was used as the blank control [13].

Following incubation at 37°C for 24 h, the effect of the extracts on the growth of *S. epidermidis* was evaluated using the microplate reader at optical density of 620 nm (OD_620 nm_). The biofilm formation of *S. epidermidis *ATCC 35984 in the presence of the herbal recipe extracts was subsequently determined using the MTT reduction assay as already stated.

### 2.4. Biofilm Time-Dependent Inhibition Assay

According to anti-biofilm inhibition, THR-SK004 ethanol extract (THR-SK004E) was selected for further experiment. The biofilm development of *S. epidermidis *after being treated with this effective extract at concentrations 250 and 500 *μ*g/mL was observed every 12 h for 2 days using the MTT reduction assay as described above. 

### 2.5. Observation on Biofilm Formation by Scanning Electron Microscopy

In addition, scanning electron microscopy (SEM) images were taken to confirm the prevention of biofilm formation by THR-SK004E [14]. Briefly, this strain was allowed to grow on squared glass slides (1 × 1 cm) placed in 24-well polystyrene plates (Greiner Bio-One, France) supplemented with TSBG1c containing the extract at 250 and 500 *μ*g/mL followed by incubation at 37°C for 48 h. After removal of the media and washing, the samples were initially fixed in 2.5% glutaraldehyde in cacodylate buffer for 90 min, then washed twice with cacodylate buffer and dehydrated for 10 min using a graded ethanol series. A critical point drying procedure was followed, and the specimens were then sputter-coated with gold. Samples were examined with a scanning electron microscope (5800LV, JEOL, Japan).

Beside, the biofilm glass pieces were washed three times with PBS and stained with 1% (w/v) crystal violet solution [15]. The biofilm formation on the stained glass pieces was dissolved with DMSO and quantified by measuring the absorbance at 620 nm using microplate reader.

### 2.6. Eradication of Established Staphylococcal Biofilm by the Herbal Recipes

Static biofilms were grown for seven days as previously described [16]. As above, the bacterial culture was prepared and incubated at 37°C for 24 h. Planktonic cells in the culture medium were then removed and fresh TSBGlc was added. This procedure was repeated daily for seven consecutive days. At the end of the 7 days of biofilm growth, the medium and the planktonic cells were gently aspirated. Thereafter, 200 *μ*L of PBS containing different concentrations of the THR-SK004E (250 and 500 *μ*g/mL as final concentrations) or THR-SK010E (10 and 20 *μ*g/mL as final concentrations) were added into the wells. Eradication of staphylococcal-preformed biofilm by the extract was measured at selected time intervals of 1, 3, 6, 12, and 24 h by the MTT reduction assay as explained above. The buffer with no antimicrobial agents was added to the positive biofilm control wells. The percentage of biofilm eradication in comparison with untreated wells was calculated using the equation [(OD_(at 0 h)_ − OD_(after treatment)_)/OD_(at 0 h)_] × 100. 

## 3. Results

*S. epidermidis* ATCC 35984 was employed as a model isolate for primary screening of the anti-biofilm ability of water and ethanol extracts prepared from the traditional herbal recipes. Extraction yields of three selected herbal recipes including THR-SK004 and THR-SK010 used for wound healing and THR-SK011 used for abscess treatment and reported biological activities of their herbal components are summarized in Table 1.

**Figure 1:**
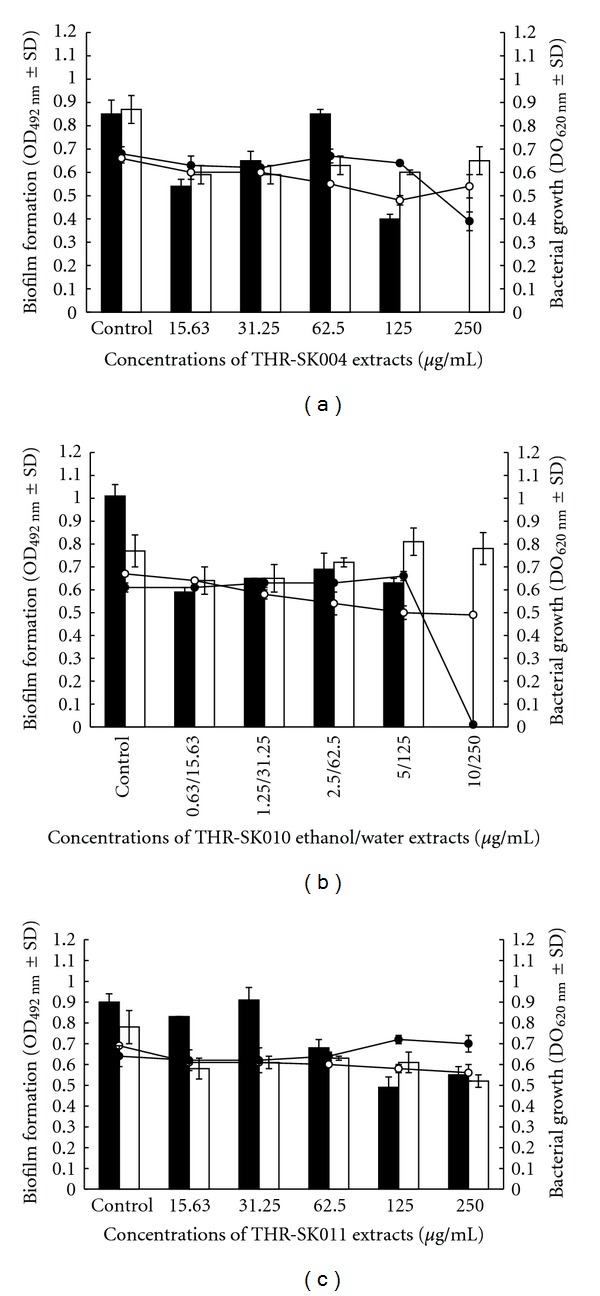
Effect of different concentrations of THR-SK004 (a), THR-SK010 (b), and THR-SK011 (c) ethanol (-*⚫*-,■), and water (-o-, □) extracts on the bacterial growth (linear charts) and the biofilm formation (column charts) of *Staphylococcus epidermidis* ATCC 35984.

**Figure 2:**
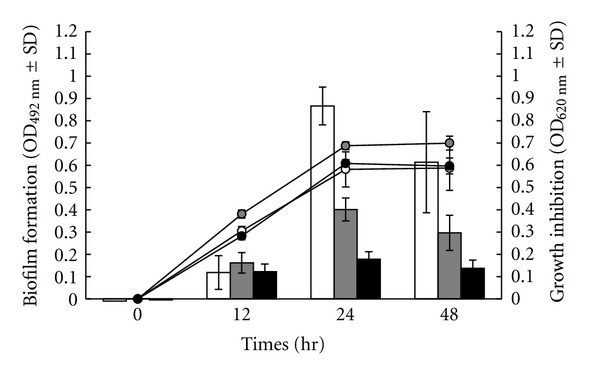
Development of *Staphylococcus epidermidis* ATCC 35984 biofilm (column charts) and the bacterial growth (linear charts) after treatment with THR-SK004 ethanol extract at 125 (grey symbols) and 250 *μ*g/mL (black symbols). Dimethylsulfoxide at 0.2% (white symbols) was used as positive control. Each symbol indicates the means ± standard error for three independent experiments performed in duplicate.

In order to investigate the effect of the recipes on *S. epidermidis* biofilm formation, the relationship between drug doses and the metabolic activity of cells in biofilm was monitored (Figure 1). The MTT reduction assay results showed that THR-SK004E at 250 *μ*g/mL could inhibit the formation of *S. epidermidis* biofilm. THR-SK010E (5–0.63 *μ*g/mL) could decrease 30 to 40% of staphylococcal biofilm. It is noteworthy that the effective concentrations of both THR-SK004E and THR-SK010E did not affect the growth of planktonic cells. Moreover, THR-SK004E at concentrations 125 and 250 *μ*g/mL was able to inhibit biofilm formation of *S. epidermidis *on polystyrene surfaces over a 48 h period as depicted in Figure 2.

**Figure 3:**
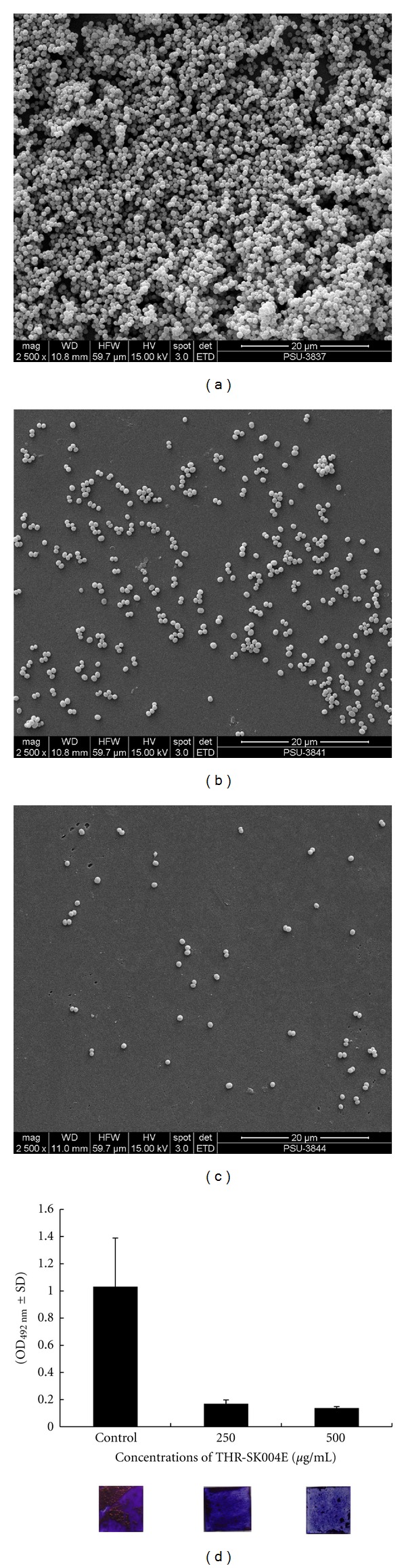
SEM micrographs of *S. epidermidis* ATCC 35984 biofilm formation on glass surfaces. Biofilms were grown in TSBGlc (a) or in TSBGlc supplemented with THR-SK004 ethanol extract at 250, (b) and 500 *μ*g/mL, (c), and all images shown were taken at magnification 2500x. The selected images were chosen as the best representatives of the amount of biofilm on the glass surfaces. Inhibition of staphylococcal biofilm development by THR-SK004 ethanol extract was additionally confirmed by crystal violet assay (d).

Inhibition of biofilm formation on glass surfaces by THR-SK004E was additionally visualized by both SEM and crystal violet assay which is illustrated in Figure 3. As expected, the additions of THR-SK004E at 250 or 500 *μ*g/mL, which reduced the staphylococcal biofilm formation on polystyrene surfaces remarkably inhibited the biofilm formation of the pathogen on glass surfaces. 

**Figure 4:**
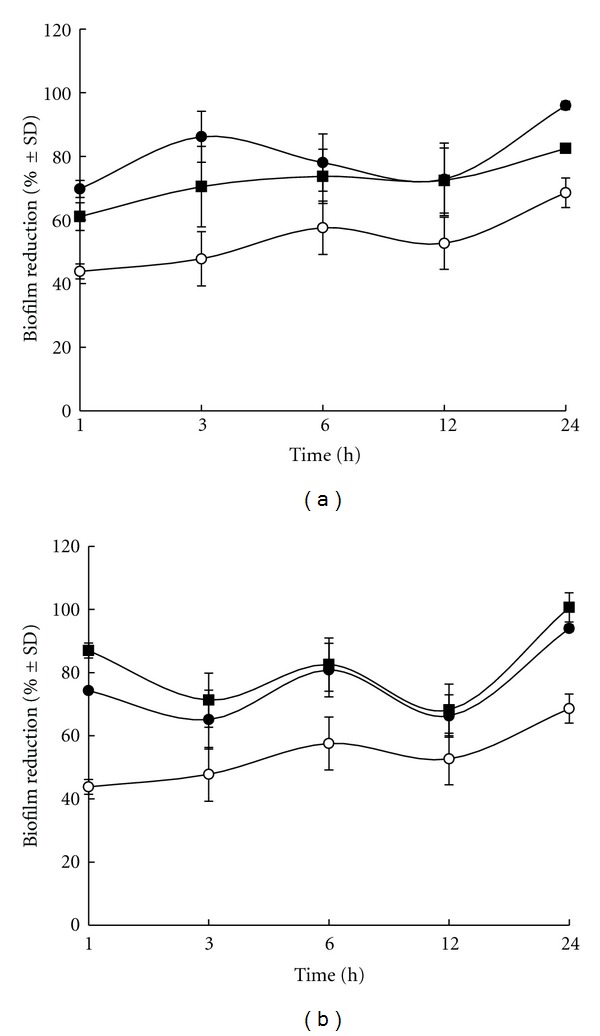
Time-dependent eradication of the mature biofilm formed by *S. epidermidis *ATCC 35984 after treatment with THR-SK004 ethanol extract (a) at 250 (*⚫*) and 500 *μ*g/mL (■) or THR-SK010 ethanol extract (b) at 10 *μ*g/mL (*⚫*) and 20 *μ*g/mL (■). Dimethylsulfoxide at 0.2% (o) was used as positive control. Each symbol indicates the means ± standard error for three independent experiments performed in duplicate.

Static *S. epidermidis *biofilm were grown for seven days and then treated with THR-SK004E (250 and 500 *μ*g/mL) and THR-SK010E (10 and 20 *μ*g/mL). As presented in Figure 4, more than 60% of the static biofilm reduction was noted in biofilms treated with the extracts for 1 h. The effect of both extracts was much more pronounced with a more lengthy treatment regimen. Noticeably, more than 90% of the 7-day-old biofilms was destroyed following a 24 h treatment with THR-SK004E at 500 *μ*g/mL and THR-SK010E at 10 and 20 *μ*g/mL. 

## 4. Discussion

Impairment of bacterial adhesion and biofilm formation by a pathway that does not influence bacterial growth is a characteristic for antivirulence therapies, one of the recent promising alternatives to combat pathogenic microorganisms, particularly *S. epidermidis* [31]. In addition to the antibacterial activity and antibiofilm potency of individual medicinal plants, the effects of herbal recipes on *S. epidermidis* biofilm were studied for the first time.

 THR-SK004 and its herbal constituents (*Maranta arundinacea*, *Oroxylum indicum*, and *Commelina benghalensis*) have never been judged for their antibiofilm ability. However, ethanol extracts of THR-SK004, *Commelina benghalensis,* and *Oroxylum indicum *were proposed to have mild-to-moderate antistaphylococcal activities [12, 32, 33]. Treatment with THR-SK004E resulted in a great inhibition of *S. epidermidis* biofilm formation, but the presence of the extract did not influence the bacterial growth. This study demonstrates that the recipe extract inhibits the formation of *S. epidermidis* on both hydrophobic surface (polystyrene) and hydrophilic surface (glass). The information suggests that intensive study on THR-SK004E active constituents may potentially be used as a tool to prevent biofilm formation on both hydrophobic and hydrophilic medicinal devices. Likewise, previous investigations have implied that coating clinical materials with antimicrobial substances successfully prevents microbial colonization and biofilm formation [10, 13, 34, 35].

Ethanol extract of THR-SK010 is composed of *Oryza sativa *and other well-documented medicinal plants including* Curcuma longa*, *Areca catechu*, and *Garcinia mangostana*. The active constituents of* Curcuma longa *have been proved or anti-biofilm and antiadherence potencies on *Candida albicans *[36]*, Streptococcus mutans *[37], *Vibrio vulnificus* [38], and *Pseudomonas aeruginosa* [39]. However, at the tested concentrations (5–0.63 *μ*g/mL) which are lower than the values used in the previous reports, THR-SK010 did not inhibit the bacterial growth but showed a slight antibiofilm activity. However, anti-biofilm staphylococcal formation activity of water extracts of the tested recipes and THR-SK011E was not demonstrated. Among the herbal components of the recipes, 12 (92.3%) of them have been reported to possess wound healing or related biological activities such as antioxidant and anti-inflammatory activities [40]. In addition, our results concur with literature evidence that ethanol is a more reliable extraction solvent of antimicrobial substances from medicinal plants compared to water, representative of the therapeutically effective preparations currently favoured by traditional healers [40, 41].

 As there is an urgent need to identify therapeutic strategies that are directed toward the inhibition of bacterial preformed biofilm, the eradication potency of effective extracts was evaluated. This present study showed that both THR-SK004E and THR-SK010E successfully eliminated the established biofilm of *S. epidermidis*, even at low concentrations of THR-SK010E (10 to 20 *μ*g/mL). Antipreformed biofilm activity of THR-SK010E is comparable with antibiotics (daptomycin, linezolid, and tigecycline) [42] or plant-derive compounds (eucalyptus oil, 1,8-cineole [43], tea tree oil [44], farnesol [16], oregano, carvacrol, and thymol [10]). 

There is a critical need for the development of alternative treatment to combat the growing number of multidrug resistant pathogen-associated infections, especially in situation where biofilms are involved. THR-SK004E strongly exhibited anti-biofilm formation of *S. epidermidis* on both polystyrene and glass surfaces, whereas both THR-SK004E and THR-SK010E remarkably destroyed the established biofilm.

## 5. Conclusion

Based on our results, THR-SK004E and THR-SK010E have promising applications as alternative antibiofilm agents. Close investigations into the identification of active constituents from the effective recipes and study on mechanisms involved in the inhibition of biofilm by the recipes are therefore warranted and currently being pursued in our laboratory. 

## Figures and Tables

**Table 1:** Wound healing related-biological activities, herbal components, and extraction yields of selected southern Thai herbal recipes.

Herbal components (Plant parts)	Yield (%)	Wound healing related-biological activities	References
THR-SK004	2.40/2.22^a^		
*Maranta arundinacea *L. (*Rhizome*)		NA	
*Oroxylum indicum* Vent. (Bark)		Ulcer protective	[17]
*Commelina benghalensis* L. (Whole plant)		Anti-oxidant	[18]

THR-SK010	6.45/3.43		
*Curcuma longa *L. (*Rhizome*)		Anti-oxidant/anti-inflammatory/wound healing	[19]
*Areca catechu *L. (Seed)		Anti-inflammatory	[20]
*Oryza sativa *L. (Seed)		NA	
*Garcinia mangostana *L. (Pericarp)		Anti-inflammatory; Anti-ulcerogenic	[21, 22]

THR-SK011	2.01/3.33		
*Ceiba pentandra* L. Gaertn. (Leaf)		Anti-oxidant; Anti-inflammatory	[23] [24]
*Aloe barbadensis* Mill. (Leaf)		Anti-oxidant/anti-inflammatory	[25]
*Coccinia grandis* (L.) Voigt. (Climber)		Anti-oxidant	[26]
*Senna siamea* (Lam.) Irwin & Barneby. (Leaf)		Anti-inflammatory	[27]
*Chromolaena odorata* (L.) R. M. King & H. Rob. (Climber)		Anti-oxidant; Wound healing	[28, 29]
*Tinospora crispa *(L.) Miers ex Hook. f. & Thomson. (Climber)		Anti-oxidant/anti-tumor	[30]

^a^Extraction yields of ethanol/water extracts.
